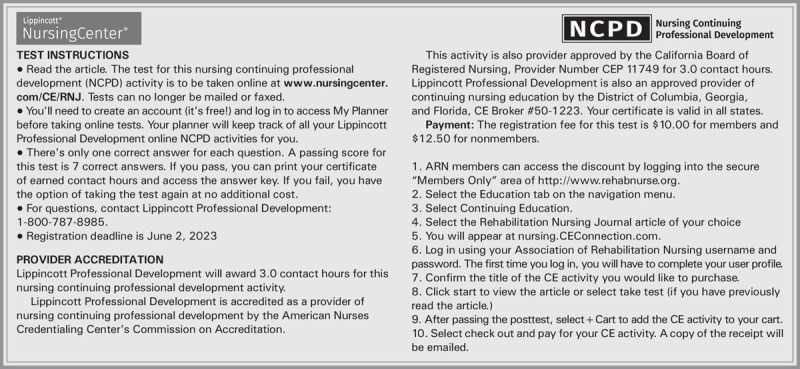# Chronic Illness and Fatigue in Older Individuals: A Systematic Review

**DOI:** 10.1097/RNJ.0000000000000278

**Published:** 2020-07-13

**Authors:** Maral Torossian, Cynthia S. Jacelon

**Affiliations:** 1 University of Massachusetts Amherst, Amherst, MA, USA

**Keywords:** Chronic disease, chronic illness, fatigue, fatigue consequences, fatigue management, fatigue risk factors, older individuals

## Abstract

**Background:**

Fatigue is a symptom experienced by 40%–74% of older individuals in the United States. Despite its significance, clinicians face challenges helping individuals to manage or reduce fatigue levels. Some management issues are attributable to the ambiguity around the risk factors, consequences, and the effect of fatigue management strategies.

**Methods:**

A literature review was conducted using four databases to identify themes in relation to risk factors, consequences, and management strategies from research studies about fatigue in older individuals with chronic diseases.

**Results:**

Findings on fatigue risk factors, such as age, body mass index, and marital status, were contradictory. There was a positive association between fatigue and comorbidities, depression, and anxiety and a negative relationship between fatigue and physical activity, sleep, educational status, and socioeconomic status. Fatigue was perceived as a state of “feebleness” and negatively impacted individuals’ quality of life. Consequences of fatigue included tiredness, sleepiness, depression, anxiety, worse sense of purpose in life, poor self-care, and an increased β-amyloid load. Predictors of worse fatigue consequences included functional health, symptom burden, subjective health, and self-acceptance. Fatigue management strategies included physical activity, rest, sleep, maintaining normal hemoglobin levels, and acetyl-l-carnitine supplementation.

**Conclusion:**

This systematic review is of value to older individuals with chronic illnesses, researchers, and clinicians who strive to improve the quality of life of individuals experiencing fatigue. To prevent undesirable consequences of fatigue, older individuals should be screened for the discussed modifiable risk factors of fatigue. The inconsistencies in the studies reviewed can guide researchers to potential research areas that require further inquiry and exploration to ground future practice on best scientific evidence

Fatigue is a cardinal symptom experienced by 40%–74% of older individuals living with a chronic disease (Menting et al., 2018). Fatigue is defined as an overwhelming sense of decreased capacity for activity, physical or mental, due to an imbalance in the availability, utilization, or restoration of resources (Aaronson et al., 1999), and is attributed to disease-specific, psychological, or cognitive factors (Goedendorp et al., 2014). Fatigue is described as an unpleasant, troublesome, and burdensome symptom, contributing to irritability, poor motivation, attention, memory, and a decline in social and physical function (Menting et al., 2018; Ream & Richardson, 1996).

Although individuals in all age groups experience fatigue, this is a concept of particular interest in older individuals. First, fatigue is one of the most prevalent symptoms reported in older individuals, whereby 77% of patients above the age of 70 years reported fatigue upon hospital admission, and had an odds ratio (*OR*) of 3.20 to retain this symptom for 3 months following discharge (van Seben et al., 2019). Second, fatigue can be one of the early signs of aging and a self-reported indicator of frailty (Avlund, 2010). Thus, advancing knowledge in fatigue-related factors and effective fatigue management strategies can help delay frailty, slow down the aging process, and reduce the odds of symptom persistence post hospital discharge. Third, fatigue is an independent predictor of mortality in this population, whereby individuals with hematological malignancies who experienced more fatigue had significantly worse prognosis and a shortened overall survival, compared with those with lower levels of fatigue (Hofer et al., 2018). In addition, fatigue becomes more debilitating and limiting when it coexists with chronic conditions, a phenomenon common in older adult. Yet, fatigue is often viewed as a normal part of the aging process, rather than a manifestation of an underlying condition.

Geriatric syndromes are a cluster of multifactorial disease presentations that are nonspecific, common across many diseases, and linked to common risk factors (Inouye, Studenski, Tinetti, & Kuchal, 2007). To date, the classification of fatigue as a geriatric syndrome is vague. Some studies have included fatigue when examining the prevalence or progression of geriatric syndromes (van Seben et al., 2019), whereas others have examined cognitive and functional decline, weight loss, incontinence, falls, and depression, without including fatigue (Bell et al., 2016; Tang, Tang, Hu, & Chen, 2017). This, along with unclear causes of fatigue and treatment strategies, adds to the confusion about the concept of fatigue.

Currently, there is an increased interest in chronic disease symptom management, including fatigue. Many research studies have addressed fatigue in terms of its triggers, consequences, management strategies, older individuals’ perceptions of fatigue, and so forth. However, clinicians face challenges understanding the risk factors of fatigue and effective treatment strategies. Study findings either pertain to a single chronic disease, a geographic location, or an age group, which renders them less useful for application in practice. Systematic reviews comparing and contrasting findings of different studies are needed to identify themes across studies. Literature reviews conducted between 2013 and 2018 included supportive care measures in older individuals with cancer (Naeim, Aapro, Subbarao, & Balducci, 2014), the contribution of occupational and physical therapy self-management interventions in chronic disease (Richardson et al., 2014), and experiences of older individuals with heart disease (Falk, Ekman, Anderson, Fu, & Granger, 2013). However, to date, there has not been a review focused on findings across studies that address risk factors of fatigue, consequences, and fatigue management strategies.

Understanding risk factors of fatigue that are common across multiple chronic diseases may lead to the development of fatigue management interventions applicable to more than one chronic disease—a transdiagnostic approach—and thus benefit a wider scope of older individuals with various chronic diseases while being aware of disease-specific triggers that require tailored interventions. Furthermore, evaluating the effectiveness of current fatigue management strategies is important to address gaps and guide future research in this area to improve existing interventions. More effective fatigue intervention, in turn, would improve fatigue levels experienced by older individuals and enhance their quality of life. Hence, the purpose of this literature review was to answer the following research questions: (1) What is the current state-of-art regarding risk factors and consequences of fatigue in older individuals with multiple chronic illnesses? (2) How is fatigue perceived by those experiencing it, and how does it impact their lives? (3) What are the current fatigue management interventions in this population?

## Methods

To achieve the purpose of the study, a systematic literature review was conducted using the following keywords: “fatigue” (field: title), “older adults or geriatrics or seniors or elderly” (field: text), and “chronic disease or chronic condition or chronic illness or long-term condition” (all fields). Four databases (CINAHL, PubMed, PsychInfo, and Web of Science) were searched with the following restrictions if the option was provided in the database: peer-reviewed (not an option in PubMed), English language, and sample age of 65 years or older. No year restrictions were applied, as the aim of this study was to capture the evolution of findings across time. Following the search process, each abstract was read by both researchers. Those saved met the inclusion criteria at this point: primary sources, peer-reviewed, English language, title included “fatigue” and its relation to a comorbidity/chronic disease, and had a mean sample age of 65 years or older (or ran a separate analysis of this age group). Articles were excluded if they were secondary sources (literature reviews), had a mean sample age of less than 65 years, or were irrelevant to the question of interest. That is, if studies addressed fatigue in relation to variables other than chronic diseases, they were excluded from the study. There were no specific diseases or research methodologies determined a priori for inclusion, as the goal was to gather qualitative and quantitative data from the widest range of chronic diseases in which fatigue was a commonly reported symptom in an older adult.

The researchers then reread, categorized, and grouped the final number of articles to be included in the review based on the aims/topics addressed. The articles were organized into a matrix (Garrard, 2017). The matrix included individual study characteristics, including author, year, study design, type of chronic illness, sample age, sample size, study aim, measurement of fatigue, risk factors, consequences, perceptions, and management of fatigue (Table 1). The categories of the matrix were used to guide the appraisal of each article, which was conducted by the first author and reviewed by the second author. A consensus about the final number of articles was reached following detailed discussions about the characteristics of each of the articles. Articles were also assessed for biases and limitations, which are presented throughout the article.

**Table 1:** Characteristics of the Articles Included in the Systematic Review

Review Article Author (Year), Location	Design	Chronic Illness of Sample	Sample Age (years)	Sample Size	Aim	Fatigue Measurement Tool
Agnihotri et al. (2007), IL, USA	Double-blinded RCT, crossover design	Chronic anemia, in addition to HTN, hypercholesterolemia, DM (Type 2), CHF, CAD, gout	Mean age: 76.1	*N* = 54	Evaluate the effect of epoetin alfa treatment on hemoglobin, fatigue, quality of life, and mobility in elderly patients with chronic anemia	FACIT-An
Ekman & Ehrenberg (2002), Sweden	Descriptive	Chronic heart failure	Women: 83 Men: 78	*N* = 158	Describe and compare the experience of fatigue in a group of elderly women and men with severe chronic heart failure	Modified version of Fatigue Interview Schedule VAS
Galindo-Ciocon & Ciocon (1997), FL, USA	Cross-sectional	Obesity, cardiac, HTN, neurologic, lung disease, sleep disorders, fibromyalgia, arthritis, depression	Mean age: 72	*N* = 83	Objectively measure chronic fatigue and identify factors that contribute to its occurrence in older adults	CFS
Hägglund et al. (2008), Sweden	Explorative, descriptive	Chronic heart failure	73–89	*N* = 10	Illuminate the lived experience of fatigue among elderly women with CHF	N/A
Hardy & Studenski (2010), PA, USA	Cross-sectional	Cardiovascular, neurological, musculoskeletal, pulmonary, diabetes, cancer, visual, and general (depression, sleep problem, chronic pain etc.)	Mean age: 74	*N* = 495	Identify the qualities of fatigue and assess whether they are associated with distinct chronic conditions	Candidate questions chosen from literature for 5 fatigue qualities
Hawker et al. (2010), Canada	Cross-sectional	OA	Mean age: 78	*N* = 613	Evaluate the relationship between subjective sleep quality and fatigue in individuals with OA	POMS-F
Hooper et al. (2017), France	Cross-sectional	Individuals with memory complaints and difficulty performing ADL	Median age: 75	*N* = 269	Explore the cross-sectional relationship between fatigue and cerebral β-amyloid in 269 elderly individuals	2 Likert-scale questions
Horne et al. (2019), NC, USA	Cross-sectional	CVD and other comorbidities	Mean age: 76	*N* = 98	Examine comorbidity measures that may relate to the symptom of fatigue post MI	RPFS - Revised Piper Fatigue Scale
Jing et al. (2015), China	Cross-sectional	Not mentioned	60–74: 390/1,272 ≥ 75: 144/1,272	*N* = 534 Total *N* = 1,272	Investigate the prevalence of fatigue, explore the relationship between gynecological history and experiences of fatigue, and identify risk factors for fatigue in middle-aged and elderly women	CFS
Kapella et al. (2006), IL, USA	Cross-sectional	COPD	Women: 68.7 Men: 69.5	*N* = 130	(a) Describe characteristics of fatigue in people with COPD and (b) test a theoretically and empirically supported model of the relationships among subjective fatigue, dyspnea, functional performance, anxious and depressed moods, and sleep quality in people with COPD	Numerical Rating Scale for fatigue dimensions Fatigue Assessment Instrument for COPD-related fatigue
Karakoc & Yurtsever (2010), Turkey	Descriptive	Cancer	60–64: 31/71 ≥ 65: 40/71	*N* = 71	Determine the relationship between fatigue and social support in elderly individuals receiving chemotherapy	VAS-F
Karakurt & Ünsal (2013), Turkey	Cross-sectional	COPD and other chronic conditions (heart disease, DM, hyperlipidemia, HTN, OA, etc.)	Mean: 68.87	*N* = 255	Determine the fatigue, anxiety and depression levels, activities of daily living of patients with chronic obstructive pulmonary disease	VAS-F
Kessing et al. (2016), the Netherlands	Secondary analysis of prospective data from 2 studies	CHF	Mean age: 66.2	*N* = 545	Examine whether general and exertion fatigue are distinctively associated with self-care in patients with chronic HF	Fatigue Assessment Scale Dutch Exertion Fatigue Scale
Lin et al. (2015), China	Cross-sectional	Not specified	≥65: 304/1,158	*N* = 1158	Explore the prevalence of fatigue and identify the risk factors of fatigue among men aged 45 years and older in China	CFS
Malaguarnera et al. (2008), Italy	Double-blinded RCT	Not specified	Women: 76.2 Men: 78.4	*N* = 96	Evaluate the effect of exogenous ALC on the physical functions and cognitive status in elderly patients with fatigue	Wessely and Powell score Fatigue Severity Scale
Mollaoglu et al. (2011), Turkey	Descriptive	COPD	Mean age: 72	*N* = 98	Describe the prevalence and severity of fatigue and to investigate relationships between fatigue and disability in elderly COPD patients	VAS-F
Muszalik et al. (2016), Poland	Survey	Breast cancer	>61: 42/120	*N* = 120	Assess the quality of life in women undergoing radiotherapy for the treatment of breast cancer	FACIT-F
Nicklas et al. (2016), NC, USA	Cross-sectional and longitudinal design	Arthritis, HTN, CVD, DM, osteoporosis, cancer	Mean age: 66.2	*N* = 167	Determine the cross-sectional and longitudinal relationships of objectively measured habitual PA to biomarkers of inflammation and self-reported fatigue in middle-aged and older adults	SF-36 Vitality Subscale of Medical Outcomes Study
Plach et al. (2006), WI, USA	Secondary analysis of data from cross-sectional study	HF	Mean age: 69	*N* = 169	Describe representations of one HF-related symptom, fatigue, and examine whether representations were related to physical health status, healthcare utilization, and psychological well-being and whether they differed by age	Symptom Representation Questionnaire for Fatigue
Silva et al. (2011), Brazil	Observational, cross-sectional	Not mentioned	Mean age: 71.29	*N* = 135	Investigate the association of clinical, functional, and inflammatory factors with muscle fatigue and self-perceived fatigue in elderly women	VAS-F
Stephen (2008), ID, USA	Cross-sectional	HF	Mean age: 77	*N* = 53	Describe the relationships between fatigue intensity and symptom experience and symptom outcomes (functional status, quality of life, satisfaction) Identify the demographic, clinical, and symptom outcome predictors of fatigue intensity in older adults with stable HF	POMS-F VAS-F
Theander & Unosson (2011), Sweden	Descriptive cross-sectional	COPD	N/A	*N* COPD = 345 *N* Control = 245	Examine gender differences in experiences of fatigue and functional limitations due to fatigue in patients with chronic obstructive pulmonary disease and a comparison group	Structured questions about fatigue frequency, severity, and duration

*Note.* RCT = randomized controlled trial; HTN = hypertension; DM = diabetes mellitus; CHF = chronic heart failure; OA = osteoarthritis; ADL = activities of daily living; COPD = chronic obstructive pulmonary disease; CFS = Chalder Fatigue Scale ; POMS-F = Profile of Mood States Fatigue Subscale; PA = physical activity; SF-36 = Short Form-36 item; CAD = coronary artery disease; CVD = cardiovascular disease; HF = heart failure; NA = not applicable; FACIT-An = Functional Assessment of Chronic Illness Therapy -Anemia; VAS = visual analog scale; VAS-F = Visual Analog Scale -Fatigue; ALC = Acetyl-L-Carnitine; FACIT-F = Functional Assessment of Chronic Illness Therapy-Fatigue.

## Results

**Figure 1:**
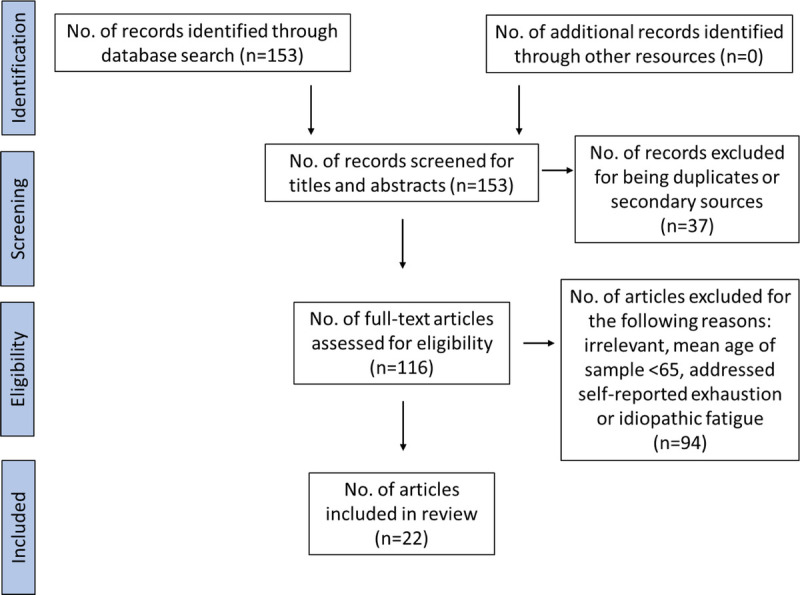
Preferred Reporting Items for Systematic Reviews and Meta-Analyses flowchart of the articles included in the review.

The search strategy yielded 153 articles across all four databases. Of these, 37 studies were excluded for being duplicates or secondary sources, and 116 articles were saved. After reexamining the 116 articles, 94 were further excluded for having a mean sample age of less than 65 years or addressing fatigue in relation to variables other than a chronic disease (sleep, pain, poststroke fatigue, fatigue in caregivers, self-reported exhaustion, or idiopathic fatigue). The final number of research studies that met the inclusion criteria and were included in the review was 22. The authors followed the Preferred Reporting Items for Systematic Reviews and Meta-Analyses (PRISMA) reporting guideline throughout the article and used the PRISMA flowchart to summarize the steps taken throughout the search process (Moher et al., 2015; Shamseer et al., 2015; see Figure 1). Narrative synthesis was used to synthesize the findings (Arai et al., 2007; Rodgers et al., 2009), a commonly used approach in systematic reviews when statistical meta-analysis of effectiveness data is not possible due to heterogeneity of studies.

### Sample Characteristics

Research studies included in the review were focused on fatigue in older individuals diagnosed with a broad range of chronic diseases, the most common of which were chronic obstructive pulmonary disease (COPD), congestive heart failure, and cancer. Sixteen of the 22 studies addressed risk factors, and nine addressed consequences, individual perceptions, or management as well. One study focused on consequences, two on subjective perceptions and impact on daily life, and two discussed fatigue management. One study addressed both impact of fatigue on daily life and management strategies. The research sites of studies included seven from the United States, whereas the remaining studies were conducted in European countries.

### Risk Factors of Fatigue

Sixteen studies in the sample were focused on the first research question: the relationship between fatigue and different variables as risk factors. These studies included biophysiological, sociodemographic, psychological, and lifestyle risk factors.

#### Biophysiological Factors

These included age, body mass index (BMI), comorbidities, gender-related factors, and sleep. Age significantly correlated with muscle fatigue (*r_s_* = −.26, *p* < .01) in two studies including men or women exclusively (Lin et al., 2015; Silva et al., 2011). However, two studies recruiting women with breast cancer had conflicting findings regarding the age–fatigue association. In one study, women in the 61–70 years age group had the highest fatigue scores compared to younger or older women (Muszalik, Kolucka-Pluta, Kedziora-Kornatowska, & Robaczewska, 2016), whereas in another study, women over the age of 75 years were nearly 5 times more likely to experience fatigue (*OR* = 4.81; Jing, Wang, Lin, Lei, & Wang, 2015). A third study revealed that women between the ages of 40–64 years experienced significantly higher emotional distress secondary to fatigue, in addition to more severe fatigue consequences, when compared with women 65 years of age or older (Plach, Heidrich, & Jeske, 2006). Finally, both in men and women with heart failure, there was no correlation between fatigue intensity and age. However, subjective perceptions of age-relatedness of experienced fatigue was positively associated with fatigue intensity (Stephen, 2008).

Findings on the BMI–fatigue association were also contradictory. In two studies, individuals with higher BMI had higher fatigue scores (Lin et al., 2015; Silva et al., 2011), whereas results of another study revealed that only underweight women experienced significantly worse fatigue compared to other groups (normal, overweight, obese; Jing et al., 2015). It is important to mention that, in the former study, BMI–fatigue correlation was insignificant in a multivariate linear regression, when accounting for other variables (Silva et al., 2011).

Number of comorbidities was another variable examined in multiple studies; however, findings did not align either. In two studies, results showed no influence of comorbidity on fatigue (Galindo-Ciocon & Ciocon, 1997; Karakoc & Yurtsever, 2010), whereas findings in six other studies reflected the opposite. There was a significant difference in the number of comorbidities between fatigued and nonfatigued individuals (Hardy & Studenski, 2010; Horne, Johnson, & Crane, 2019; Lin et al., 2015) and a positive correlation between the number of comorbidities and perceived fatigue (*r_s_* = .18, *p* < .05; Silva et al., 2011). Interestingly, self-reported comorbidities explained 9% of the variance in fatigue scores in women but was not a significant predictor in men (Horne et al., 2019). Yet, the experience of concurrent symptoms caused by heart failure or other comorbidities, both in men and women, was significantly correlated with fatigue intensity (Stephen, 2008). Lastly, women with breast cancer who had one comorbidity had 1.83 times higher risk of fatigue, and this risk was threefold with two or more chronic diseases (Jing et al., 2015). This discrepancy may be due to a number of factors. First, two studies (Galindo-Ciocon & Ciocon, 1997; Karakoc & Yurtsever, 2010) recruited both men and women as opposed to most of the other studies, in which only men, or women, were recruited. Second, the number of comorbidities varied between and within studies, making a significant difference undetectable when two groups were similar in terms of this variable. Lastly, these studies used different fatigue measure tools, reflecting different dimensions of fatigue, and measured different aspects of fatigue (risk of fatigue, fatigue intensity, perceived fatigue).

Of the women-specific variables, results of a study on women with breast cancer revealed that the number of live births significantly correlated with fatigue, and the odds of fatigue was 4.17 times higher in women who had four or more live births compared to those who only had one. In addition, postmenopausal women were 1.70 times more likely to experience fatigue than premenopausal ones (Jing et al., 2015). Although the study had adequate power, and a reliable, culture-sensitive tool was used to measure fatigue (Chalder Fatigue Scale), findings should be interpreted in caution, as the sample consisted of patients with cancer only.

Finally, two studies addressing sleep found a positive correlation between sleep and fatigue. In patients with COPD, researchers found a moderate and a positive correlation between sleep (higher scores reflecting worse sleep quality) and fatigue (*r* = .4, *p* < .001; Kapella, Larson, Patel, Covey, & Berry, 2006) and worse fatigue levels in individuals with osteoarthritis who experienced poor sleep (Hawker et al., 2010). A study comparing fatigued and nonfatigued individuals found that sleep disorders were significantly more prevalent in individuals in the former group (Galindo-Ciocon & Ciocon, 1997). However, a limitation of these studies is that variables such as daily medications, number and type of comorbidities, and social support that would have influenced or mediated this relationship were not included in either of the studies.

#### Sociodemographic Factors

There was a significant negative correlation between social support (information, security, emotional, and perceived) and fatigue (−.78 < *r* < −.65, *p* < .001) and a significant positive correlation between social support and energy (.71 < *r* < .82, *p* < .001) scores (Karakoc & Yurtsever, 2010). Education and economic status were also correlated with fatigue in all but one study. Five studies showed that individuals with higher levels of education had significantly lower fatigue levels (Jing et al., 2015; Karakurt & Ünsal, 2013; Kessing, Denollet, Widdershoven, & Kupper, 2016; Lin et al., 2015; Muszalik et al., 2016); however, that did not hold true in the study by Karakoc and Yurtsever (2010). Economic status was positively associated with fatigue scores: Employed individuals or those in a “very good” economic status had significantly less fatigue compared with those unemployed (Kessing et al., 2016) or those in “good” or “difficult” economic standing (Muszalik et al., 2016).

Findings on marital status varied greatly. Two studies involving men and women showed no significant difference in fatigue scores between married, widowed, single, or divorced individuals (Horne et al., 2019; Karakoc & Yurtsever, 2010). This was contrary to findings of two studies in which married older individuals experienced significantly higher fatigue than unmarried ones (Mollaoglu, Fertelli, & Tuncay, 2011; Stephen, 2008). However, two studies involving either men or women exclusively showed that single men and single women have higher odds of fatigue (*OR* = 1.94 and *OR* = 1.42, respectively) compared with their married counterparts (Jing et al., 2015; Lin et al., 2015). To add up to the inconsistency, widowed individuals in a study experienced worse fatigue compared to married and unmarried individuals, with no differences between married and unmarried individuals (Karakurt & Ünsal, 2013).

#### Psychological Factors

Findings across multiple studies were consistent in terms of the relationship between fatigue and psychological variables. Depression and anxiety were weakly but significantly correlated with fatigue scores (*r* = .36 and *r* = .32, respectively; Karakurt & Ünsal, 2013). This correlation was supported, and even stronger, in another study as well (*r*_fatigue/depression_ = .45, *r*_fatigue/anxiety_ = .49, *p* < .001; Kapella et al., 2006). Similarly, weak but significant correlations were found in the study by Silva et al. (2011), where fatigue was positively correlated with depression (*r_s_* = .38, *p* < .01) and negatively correlated with perceived health (*r_s_* = −.25, *p* < .01). In other words, individuals with fatigue had significantly higher depression scores and poorer perceived health (Hardy & Studenski, 2010).

#### Lifestyle and Fatigue

A number of studies investigated the influence of physical activity (PA) or exercise on fatigue. A study examining the correlation of inflammation, PA, and fatigue cross-sectionally and longitudinally found a significant correlation, at baseline, between PA (measured in steps/day) and fatigue (*r* = .19, *p* < .05), but not between fatigue and inflammatory markers like C-reactive protein and interleukin-6 (Nicklas et al., 2016). The correlation between fatigue and PA remained significant at the 6-month and 18-month follow-up period, indicating that increasing activity at any point resulted in reduced fatigue levels. Interestingly, fatigue was significantly correlated with C-reactive protein and interleukin-6 at the 6-month follow-up (*r* = −.28 and *r* = −.29 respectively), but not at the 18-month follow-up (Nicklas et al., 2016). Similarly, other studies supported the correlation between PA and muscle fatigue (*r_s_* = .29, *p* < .01), perceived fatigue (*r_s_* = −.38, *p* < .01; Silva et al., 2011), and total fatigue scores (Galindo-Ciocon & Ciocon, 1997; Lin et al., 2015). Another study demonstrated that activities of daily living were negatively correlated with fatigue scores (*r* = −.45; Karakurt & Ünsal, 2013). Note that the relationship between PA and fatigue is bidirectional. That is, fatigue levels, in their turn, also impact PA. This was supported in a study in which individuals who experienced three or more qualities of fatigue showed significantly worse physical performance compared to those who only reported one fatigue quality (Hardy & Studenski, 2010).

These findings should be interpreted in light of the studies’ limitations, which included the use of a single fatigue scale, one being the Short Form-36 item Vitality subscale, which is not specific to older individuals (Nicklas et al., 2016). Another limitation is the lack of a reliable PA measure used in two of these studies (Galindo-Ciocon & Ciocon, 1997; Lin et al., 2015) and no reporting of the psychometric properties of fatigue or PA measurement tools in most studies. Finally, none of the study designs were randomized controlled trials (RCTs), meaning that causation cannot be implied.

Another lifestyle factor addressed in only one study was medication use. Diuretics, nitrates, and psychotropic medications were associated with worse fatigue scores (general and exertional), whereas exertional fatigue scores were better (less fatigued) in individuals taking β-blockers. Other medications such as Angiotensin Converting Enzyme (ACE) inhibitors, statins, aspirin, and calcium antagonists did not significantly impact fatigue scores (Kessing et al., 2016). Doses of these medications were not mentioned, making inferences for medication management of fatigue reduction impossible.

### Consequences of Fatigue

In an attempt to identify qualities of fatigue and examine their association with distinct clinical characteristics, Hardy and Studenski (2010) conducted a research study with 495 older adults diagnosed with various chronic diseases. Participants were asked to complete surveys related to the number of chronic conditions, self-rated health, physical function/performance, depression, and presence/absence of fatigue qualities. The researchers identified fatigue qualities based on the fatigue measure tools in the literature including the Cancer Fatigue Scale, the Revised Piper Fatigue Scale, the Schwartz Cancer Fatigue Scale, the Multidimensional Fatigue Inventory, and others. Qualities of fatigue were categorized as mental (consisting of emotional and cognitive domains) or physical (relating to weakness, loss of energy, and sleepiness). The most commonly reported fatigue quality was tiredness, and the least reported was emotional fatigue, with sleepiness and tiredness being significantly more prevalent in women than in men. Results showed small to moderate correlations between fatigue qualities, suggesting that each represented a distinct underlying pathophysiology. However, there was overlap between distinct conditions and their associated fatigue qualities. For example, pulmonary, musculoskeletal, cardiovascular, and neurological disorders had higher odds of experiencing sleepiness and tiredness (two qualities of fatigue), despite being linked to different pathophysiological processes. Hence, researchers concluded that qualities of fatigue represent manifestations of a common underlying process like inflammation, common across different chronic diseases (Hardy & Studenski, 2010). The researchers did not discuss details of the literature review process and how they had determined the final five qualities of fatigue. Yet, these findings can still provide a basis for clinicians in the management of the different qualities of fatigue experienced by individuals with chronic diseases.

A number of predictors were associated with the severity of fatigue consequences. For example, functional health, heart failure symptom burden (higher scores indicating less symptom burden), subjective health, purpose in life, and self-acceptance were negatively associated with severity of fatigue consequence (*r* = −.43, *r* = −.55, *r* = −.36, *r* = −.26, and *r* = −.27, respectively). Depression, anxiety, symptom burden of other health problems, and number of physician visits in the past year positively correlated with fatigue consequences (*r* = .42, *r* = .34, *r* = .40, and *r* = .31, respectively). The severity of fatigue consequence was a significant predictor of depression, anxiety, and purpose in life (*r* = .40, *r* = .20, and *r* = −.22, respectively; Plach et al., 2006). Participants in this study were diagnosed with heart failure. Yet, the fatigue scale used in the study was originally developed for use among patients with cancer. The internal consistency of subscale for fatigue consequence in this sample was .71; however, other subscales did not show adequate internal consistency.

A study including individuals aged 70 years or greater and diagnosed with dementia showed a weak positive association between fatigue (measured at the clinical examination that was closest to the positron emission tomography scan) and β-amyloid load in the hippocampus (β = 0.07, *p* = .016) in individuals with a clinical dementia rating (CDR) of 0.5 (Hooper et al., 2017). Researchers carried out a sensitivity analysis on individuals of CDR of 0.5 specifically, as this subgroup had a high risk of progressing to a dementia-related illness like Alzheimer’s disease. This association was insignificant in the multivariate regression on data from the whole sample. In addition, there was no significant association between chronic fatigue and cerebral β-amyloid load, although this was not examined separately on those with a CDR of 0.5 (Hooper et al., 2017). Another drawback of the study is the use of a fatigue measure tool that was not specifically designed to capture the physiological aspect of fatigue and consisted of only two items, despite fatigue being a primary variable of interest.

Finally, poor self-care and not consulting a healthcare provider as needed were also found to be consequences of fatigue, whereby general and exertional fatigue (secondary to activity) were significant predictors of self-care (β = 0.01, *p* = .004 and β = 0.06, *p* = .01, respectively) and consultation with a healthcare provider (β = 0.05, *p* = .04 and β = 0.05, *p* = .007, respectively). This association was significant even after accounting for covariates like age, gender, and educational level, which are known to influence these behaviors. In addition, fatigue correlated positively with physical and social disability (*r* = .45, *p* < .001), with a moderate effect size (Mollaoglu et al., 2011). However, these results relied on participants’ self-reports of self-care and disability, and hence, the risk of social desirability and recall biases may be present (Kessing et al., 2016). Besides, findings pertained to individuals with heart failure only, which is another limitation.

### Subjective Perceptions of Fatigue and Its Impact on Daily Life

Researchers in a study interviewed women between the ages of 73 and 89 years to illuminate their lived experience of fatigue and how it impacts their life. According to these women, fatigue was a state of loss of energy, during which optimal rest could not be achieved, regardless of the number of hours of sleep (Hägglund, Boman, & Lundman, 2008). Individuals also experienced unfamiliar bodily sensations like numbness and breathlessness. Fatigue was unpredictable, whereby the physical ability of those experiencing it varied tremendously throughout the day, making activity planning a challenge, and rendered them in need of others’ help. On the social level, fatigue presented a networking barrier to these individuals, which led to feelings of loneliness.

In studies involving men and women, both described fatigue as a state of “feebleness” and “listlessness” with no significant difference in fatigue ratings or levels of functional limitation (Ekman & Ehrenberg, 2002; Karakurt & Ünsal, 2013; Theander & Unosson, 2011). However, women commonly perceived fatigue as “severe,” unlike men who perceived it as “mild” (Ekman & Ehrenberg, 2002). This was further supported in three studies in individuals with COPD or heart failure, whereby women experienced more fatigue than men (Kapella et al., 2006; Kessing et al., 2016; Mollaoglu et al., 2011). Both men and women with higher ratings of fatigue intensity had a worse health-related quality of life (*r* = .53, *p* < .001), and this association was even stronger in individuals who attributed fatigue to age (Stephen, 2008). Individuals coped with fatigue by appreciating the limited yet significant independence and the fact that they were still able to perform certain tasks. Interviewed women also adjusted the frequency and the timing of their activities and occasionally accepted help from others. Some were willing to use assistive devices like wheelchairs as part of the adaptation, yet others refused it for fear of becoming less active. Instead, they consciously forced themselves to be as active as possible (Hägglund et al., 2008).

Although these findings provide important insights of the lived experience of fatigue from older individuals’ perspective themselves, it is important to account for the studies’ limitations. Participants in the first study (Hägglund et al., 2008) were women diagnosed with congestive heart failure, and hence, the transferability of findings is questionable, especially with the absence of a thorough description of the study sample. In addition, steps to ensure rigor and trustworthiness such as keeping an audit trail, member checking, or peer debriefing (Lincoln & Guba, 1985) were not explicitly reported. In regard to the study by Theander and Unosson (2011), researchers did not report the reliability and the validity of the three-item fatigue measure used.

### Management of Fatigue

People with chronic illnesses experiencing fatigue adopted various self-management strategies to overcome challenges and reduce fatigue. As discussed earlier, PA was a significant predictor of lessened fatigue, and thus, engaging in PA may be an effective way to decrease fatigue levels. Combining exercise and diet in overweight individuals resulted in less fatigue, compared to adopting either of the strategies independently (Nicklas et al., 2016). Participants in different studies also reported that their fatigue responded to rest and sleep (Ekman & Ehrenberg, 2002; Kapella et al., 2006).

Maintaining normal hemoglobin levels is also a factor in fatigue management. In a RCT, older individuals receiving epoetin alfa had higher hemoglobin levels by the end of the study compared to the placebo group. This improvement in hemoglobin levels was associated with lessened fatigue scores (Agnihotri et al., 2007). However, this study was carried out on individuals with anemia only, and thus, this intervention may not be effective in individuals with normal hemoglobin levels.

A study evaluated the impact of acetyl-l-carnitine (ALC; a member of “carnitines” known to have a role in intermediary metabolism) supplementation on fatigue levels in individuals aged 70 years or older. In this double-blinded RCT, ALC demonstrated a significant impact on fatigue, where individuals receiving 2 g of ALC twice a day experienced a significant decline in fatigue levels: a 50% decrease in prolonged fatigue after activity, a 7-point decrease in physical fatigue scores, a 3.3-point decrease in mental fatigue scores, and a decline of 22.5 points on the fatigue severity scale. This was also accompanied by a 7-point increase in functional status (Malaguarnera et al., 2008). The change in scores was significantly different between the intervention group receiving ALC and the placebo group. Baseline characteristics were similar in both groups. Researchers also reported that there were no adverse drug effects or abnormal laboratory results in either of the groups.

## Discussion

This systematic review aimed to identify risk factors and consequences of fatigue investigated to date, fatigue management interventions, as well as the perceptions of fatigue by individuals with chronic illnesses and the ways in which fatigue impacts their lives. Findings demonstrated that fatigue can be a result of disease-specific, biophysiological, socioeconomical, and psychological factors, the consequences of which include limitations on individuals’ functional status, social role, self-care, depression, anxiety, health-related quality of life, and sense of purpose in life. Interventions that could improve fatigue levels included increasing PA, getting adequate rest and sleep, maintaining normal hemoglobin levels, and ALC supplementation. Although conditions differ in their pathophysiology, the overlap between different conditions and their associated fatigue qualities, as well as the correlation between fatigue, inflammation, nutrition, and performance, suggests an underlying common pathway (Hardy & Studenski, 2010; Hofer et al., 2018). Hence, besides fatigue management interventions tailored to each individual’s needs, a transdiagnostic approach might be an effective strategy to address fatigue in different chronic conditions using similar interventions. This can help identify additional gaps in the literature and examine further areas for study.

Future research should be guided toward resolving inconsistencies in findings of research studies included in this systematic review. Clarifying the ambiguity regarding the correlation between fatigue and age, gender, and BMI would be useful to determine whether clinicians should account for age and gender in determining baseline fatigue levels and whether weight management should be part of the fatigue-specific interventions or not. A study of women with cancer found certain gynecological factors to be risk factors for fatigue. Thus, it would be of value to examine whether these findings are generalizable to women without cancer as well. Besides, there was a significant association between certain medications and fatigue and between pre-positron emission tomography scan fatigue scores and β-amyloid load in individuals with a CDR of 0.5. Hence, rigorous RCTs are needed to support the preliminary findings from these descriptive studies and provide the basis for future interventions or preventive measures.

Different fatigue measures were used in the selected research studies, with the Visual Analogue Scale–Fatigue being the most common one (five studies). Given the availability of numerous fatigue measurement tools, it would be challenging to determine which scale to use in different settings or with different cultures. Hence, a review focusing on the available fatigue measure tools, their psychometric properties, and the most convenient setting/culture for the use of each of them would be helpful in guiding researchers to choose the tool that would yield the most reliable fatigue measures.

Besides the limitations of the individual studies discussed throughout the text, this systematic review has some limitations as well. The limited number of keywords, the search of only four databases, and the exclusion of the gray literature may have excluded some articles pertaining to the discussed topic. However, strengths of the review lie in the detailed presentation of the methods used for study selection, maintaining an audit trail to keep track of the decision-making process, the appraisal of individual studies for robustness, the wide scope of chronic diseases included with no publication year restrictions, the different locations in the U.S. and European countries, and inclusion of studies with a mean sample age of 65 years or older only, so that findings are generalizable and applicable to this age group specifically.

## Conclusions

This is the first systematic review that integrates findings related to risk factors, consequences, perceptions, and management strategies of fatigue in individuals aged 65 years and older with various chronic conditions. Findings of this review are of value to individuals who share similar characteristics (age, chronic diseases) as participants of the included research studies, to healthcare providers generally, and to rehabilitation nurses specifically, who follow-up older adults postdischarge and have a major role in managing their fatigue. To prevent these undesirable consequences, older individuals should be screened for the discussed modifiable risk factors of fatigue. The inconsistencies addressed can guide researchers to potential research areas to determine the best scientific evidence. Fatigue is a burdensome symptom that affects individuals’ function, psychological well-being, and quality of life. Thus, more attention should be paid to unify the approach in care delivery across multiple disciplines to reduce fatigue to the furthest extent possible.

**Key Practice Points**

- Nurses should carefully screen individuals for identified risk factors for fatigue, as this provides baseline information and guides nurses’ fatigue management interventions.
- Nurses should acknowledge older individuals’ experiences and perceptions of fatigue and develop tailored care plans to meet their needs.
- Nurses can recommend and discuss with other healthcare team members the available fatigue management strategies and validate their effectiveness in various chronic conditions.
- Inconsistencies in findings and the lack of ongoing evaluation of fatigue measure scales should guide future research toward areas that need further exploration and validation of psychometric properties of the various fatigue measurement tools.

## Conflict of Interest

The authors declare no conflict of interest.

## Funding

The development of this publication was supported by the National Institute of Nursing Research of the National Institutes of Health under Award Number P20NR016599. The content is solely the responsibility of the authors and does not necessarily represent the official views of the National Institutes of Health.

## Figures and Tables

**Figure:**
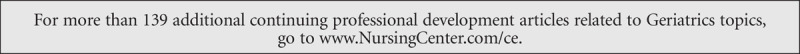


**Figure:**